# The new entries in the therapeutic armamentarium: The small molecule JAK inhibitors

**DOI:** 10.1016/j.phrs.2019.104392

**Published:** 2019-09

**Authors:** Katie Bechman, Mark Yates, James B. Galloway

**Affiliations:** Centre for Rheumatic Diseases, Kings College London, United Kingdom

**Keywords:** JAK inhibitors, Tofacitinib, Baricitinib, Upadacitinib, Rheumatic disease

## Abstract

The past decade has witnessed an explosion in trial data on JAK inhibitors (JAKi). These small molecules target the Janus kinase - signal transducer and activator of transcription (JAK-STAT) pathway, blocking crucial cytokines across a septum of rheumatic diseases. As a class, JAKi are beginning to demonstrate efficacy on par, if not superior to biologics. Two first generation JAKi are licensed for use in inflammatory arthritis; tofacitinib and baricitinib. Next-generation JAKi have been designed with selective affinity for one JAK enzymes, the aim to reduce unwanted adverse effects without declining clinical efficacy. Emerging data with selective JAK1 inhibitors upadacitinib and filgotinib looks very promising. Despite differences in selectivity between JAKi, an overlap exists in their safety profiles. Across the class, a characteristic safety signal is emerging with viral opportunistic infections, particularly herpes zoster. Post marketing drug surveillance will be essential in evaluating the long-term risk with these agents.

## 1 Introduction

Since the end of the last century, biological therapies have taken the rheumatoid arthritis (RA) pharmaceutical market by storm. Anti-TNFs were launched in the late 1990’s and have rapidly become worldwide brands. Within a decade, Humira was the highest earning product across the entire market.

The success of biologics was defined by their comparable high efficacy over traditional therapeutic agents. This was primarily driven by advances in specific target selectivity. Older treatments such as methotrexate and corticosteroids are comparatively blunt tools, with a myriad of effects across the immune system and dose limiting toxicity. Treatment with biologics has led to a seismic shift in RA managementwith a realistic goal of low disease activity or disease remission.

For the scientific community, the development of biologics has been a fortuitous process. The discovery of TNF blockade has shed light on important immune aberrancies in RA, assisting in the identification and targeting of sites in the inflammatory cascade by newer biological agents. Despite their success, biological therapies have several limitations: (1) they are expensive to manufacture even after their patents expire; (2) they require administration parenterally, as proteins they would be digested if administered orally; (3) they require a cold storage chain in their supply route; (4) they are inherently immunogenic, so can trigger the development of anti-drug antibodies.

As our understanding of the immune system in RA continues to expand, enticing targets for future immunotherapies have been identified. The drug development world for small molecular entities has been waiting in the wings and is now emerging into the limelight as a first line treatment option in RA. These small molecular inhibitors demonstrate equivalent or even superior efficacy to biologics and are free from many of their limitations.

## 2 JAK-STAT pathways

**Fig. 1:**
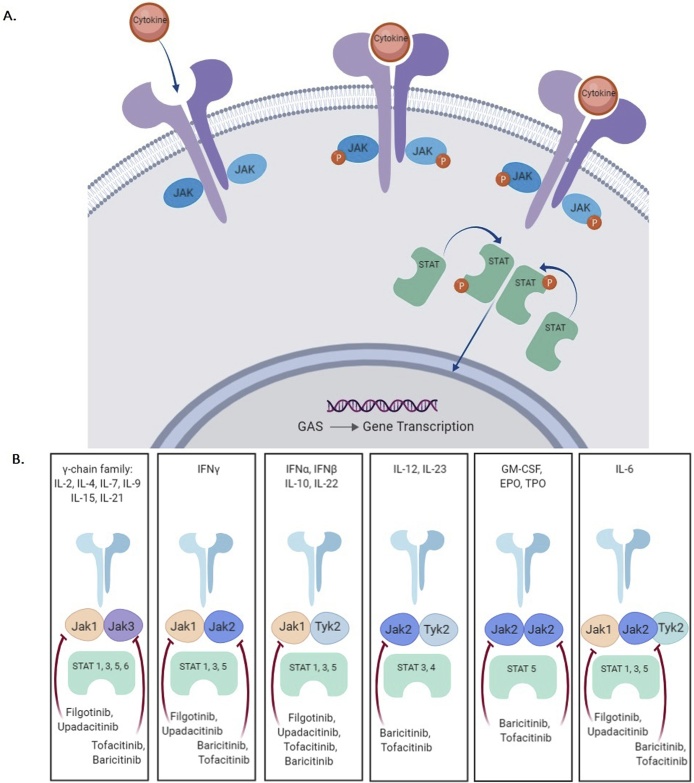
(A) The JAK-Stat signalling pathway. (B) Cytokine signally through JAK/Stat combination. Upon binding to a cytokine, the cytokine receptor associated JAKs become activated. These JAKs mediate phosphorylation of specific receptor tyrosine residues and recruited STATs. Activated STATs are released from the receptor, dimerize, translocate to the nucleus and bind to members of the GAS (Gamma Activated Site) family of enhancers. Adapted from Kisseleva el al, Gene. 2002; 285 (1-2):1–24. JAK: Janus kinase; TYK: Tyrosine kinase; STAT: Signal Transducer and Activator of Transcription; IFN: Interferon; IL: Interleukin; EPO: Erythropoietin; GM-CSF: Granulocyte/macrophage colony stimulating factor; TP0: Thrombopoietin. Adapted from Hodge et al. Clin Exp Rheumatol. 2016; 34 (2):318-328.

Janus kinases (JAKs) belong to the family of tyrosine kinases enzymes recognised by their ability to phosphorylate tyrosine residues, altering the function of the protein that they are contained in. They are able to transfer extracellular signals from cell surface receptors to the nucleus, changing DNA transcription and the subsequent translation of proteins. The JAK-STAT pathway operates downstream of more than 50 cytokines and growth factors and is regarded as a central communication node for the immune system [1]. There are four members of the JAK family: JAK1, JAK2, JAK3, and TYK2. Each cell surface receptor requires a pair of JAKs as either identical homodimers (e.g. JAK2/JAK2) or heterodimers (e.g. JAK1/JAK3) in order to signal [2]. This is in turn activates STAT proteins (signal transducers and activators of transcription), which as their names suggests target gene promoters to activate transcription [3]. Each pair of JAKs have different activating ligands and downstream effector actions (Fig. 1).

### 2.1 Malfunctioning of the JAK-STAT pathways

There are a number of human models for malfunctioning of the JAK-STAT pathway. The most dramatic evidence for how critical this pathway is comes from patients with severe combined immunodeficiency (SCID). This primary immunodeficiency results in recurrent severe infections and failure to thrive. A patient with SCID was identified as having a mutation in JAK3 in which a single amino acid substitution prevented JAK3–receptor interaction. This blocked a range of cytokine stimuli and severely impacted T cell, NK, and B cell development and function [4,5]. Negative mutations in STAT3 result in hyperimmunoglobulin E syndrome (Job’s syndrome), characterized by recurrent cutaneous and sinopulmonary bacterial infections. STAT3 mediates signalling through several classes of receptor and is essential for the production of 1L-17 and the subsequent recruitment of neutrophils, explaining the abnormalities seen with this disorder. Conversely, several myeloproliferative diseases are driven by an activating mutations of JAK2, which is crucial for downstream signal transduction of erythropoietin and thrombopoietin [5].

### 2.2 JAK inhibitors

The development of small molecules which inhibit the JAK enzymes (JAK inhibitors; JAKi) has the potential to be a game changer. These agents are beginning to demonstrate efficacy across a septum of rheumatic diseases, with results that have not been seen since the launch of TNF inhibitors. As a class they are on par if not superior to biologics in their efficacy. Additionally, they are orally administered, demonstrate a rapid onset of action and in the case of an adverse event, their short half-life allows rapid reversal of immunosuppressive effects.

## 3 The JAK inhibitors on the market

There are currently two licenced small molecule JAK inhibitors in rheumatic diseases; tofacitinib and baricitinib. These agents block more than one JAK enzyme and prevent the signalling of multiple cytokines. The inhibition profiles are however dose dependent. At higher doses both tofacitinib and baricitinib can block other members of the JAK family and lead to 'pan-JAK' inhibition [[6], [7], [8]].

### 3.1 Tofacitinib

Tofacitinib (Xeljanz®, formerly designated CP 690,550) was developed by Pfizer and became the first JAK inhibitor to be approved for the treatment of RA by the Food and Drug Administration (FDA) in 2012. The European Medicines Agency (EMA) approved tofacitinib in 2017. In the treatment of psoriatic arthritis (PsA), tofacitinib was approved by the FDA in 2017 and EMA in 2018. The FDA declined approval for psoriasis on issues of clinical efficacy and long-term safety.

#### 3.1.1 Pharmacokinetics

Tofacitinib was originally described as a selective JAK3 inhibitor. It blocks JAK3 and JAK1, with some affinity for JAK2 and limited affinity for TYK2. [9]. Consequently tofacitinib potently inhibits signalling of γc chain cytokines (IL-2, IL-4, IL-7, IL-15 and IL-21) via JAK3; but also blocks IFN-γ and IL-6 via JAK1-JAK2; and to a lesser extent IL-12 and IL-23 via JAK2-Tyk2 [9,10].

Tofacitinib is metabolised and cleared by the liver (70%) and kidneys (30%). Metabolism is primarily facilitated by CYP3A4 with minor contribution from CYP2C19 [6]. Exposure is decreased when co-administered with potent CYP inducers (e.g. rifampicin), whilst exposure is increased when co-administered with potent inhibitors of CYP3A4 (e.g. ketoconazole) or CYP2C19 (e.g. fluconazole). The dose of tofacitinib should be reduced to 5 mg once daily in patients co-prescribed CYP3A4 or CYP2C19 inhibitors [11] or in patients with severe renal impairment (creatinine clearance <30 mL/min) [12].

#### 3.1.2 Trial evidence across diseases

i) Rheumatoid arthritis

Tofacitinib has demonstrated significant efficacy in phase II and III randomized controlled trials (RCTs) in adult RA patients, both in combination with conventional synthetic disease modifying anti-rheumatic drugs (csDMARDs) including methotrexate, and as a monotherapy. Seven phase III RCTs have been conducted; ORAL Solo, Start, Sync, Step, Scan, Standard and Strategy [[13], [14], [15], [16], [17], [18], [19]] (Table 1). The most substantial body of real-world evidence comes from the US, where tofacitinib has been available since 2012. Results from the US Corrona RA registry reported patients initiating tofacitinib had longer disease duration and greater exposer to previous TNFi or biologic DMARDs (bDMARDs) than patients initiating a traditional bDMARD [20]. There was no difference in efficacy between tofacitinib monotherapy and tofacitinib combination therapy or TNFi combination therapy in bDMARD experienced patients [21]. A retrospective cohort study of patients using data from the Commercial Claims and Encounters database in the US reported similar efficacy rates at 1-year between tofacitinib and non-TNF biologics [22] An observational Japanese study reported 60% of patients achieved ≥50% improvement in clinical disease activity index (CDAI), with remission rates higher in biologic-naïve patients [23].The Swiss Clinical Quality Management registry (SCQM) also reported high rates of low disease activity and remission [24]. Crude drug retention rates were similar for tofacitinib as other biologics, with a lower risk of drug discontinuation compared to TNFi [25].

**Table 1:** Tofacitinib published pivotal phase III RCTs.

**Tofacitinib Pivotal Phase III RCTs**			
**Rheumatoid arthritis**			
ORAL Solo [13]	DMARD/biologic-IR (n = 611) 6-months	Tofa or PBO	At 3 m; significant improvement ACR20, ACR50, ACR70, HAQ-DI. Percentage with DAS28 < 2.6 was not significantly higher.
ORAL Start [14]	MTX-naive RA (n = 958) 24-months	Tofa or MTX	At 6 m; ACR70 significantly higher 5 mg & 10 mg v MTX (26%, 38% v 12%) Changes in mTSS from baseline significantly smaller (although modest).
ORAL Sync [15]	DMARD-IR (n = 792) 12-months	Tofa or PBO, with csDMARDs	At 6 m; significant improvement ACR20 & percentage with DAS28 < 2.6. At 3 m significant improvement in HAQ-DI.
ORAL Step [16]	TNF inhibitors-IR (n = 399) 6-months	Tofa or PBO, with MTX	At 3 m; significant improvement ACR20, HAQ-DI & percentage with DAS28 < 2.6.
ORAL Scan [17]	MTX-IR (797) 24-months	Tofa or PBO, with MTX	At 6 m; changes in mTSS from baseline significantly smaller with 10 mg dose only.
ORAL Standard [18]	MTX-IR (n = 717) 12-months	Tofa or ADA 40 mg or PBO, with MTX	At 6 m; ACR20 significantly higher 5 mg, 10 mg, ADA v MTX (52%, 53%, 47% v 28%). Superior efficacy to placebo. Numerically similar to ADA.
ORAL Strategy [19]	MTX-IR (n = 1146) 12-months	Tofa +/- MTX, or ADA or PBO, with MTX	At 6 m; ACR50 were 38% tofa, 46% tofa/MTX & 44% ADA/MTX. Non-inferiority for tofa/MTX V ADA but not tofacitinib monotherapy.

**Psoriatic arthritis**			
ORAL Broaden [26]	DMARD -IR (n = 422) 12-months	Tofa or ADA 40 mg or PBO, with csDMARDs	At 3 m; ACR20 significantly higher 5 mg, 10 mg, ADA v PBO (50% 61% 52% v 33%). Improved HAQ-DI, ACR50, ACR70, PASI75 both doses & LEI with 10 mg.
OPAL Beyond [27]	TNF inhibitors-IR (n = 395) 12-months	Tofa or placebo, with csDMARDs	At 3 m; ACR20 significantly higher 5 mg, 10 mg v PBO (50% 47% v 24%). Improved HAQ-DI, ACR50 (not ACR70) both doses & PASI75 with 10 mg.

**Psoriasis**			
OPT Pivotal 1 [31]	cs-therapy-IR (n = 1106) 12-weeks	Tofa or ETN 50 mg or PBO	At 12 w; PASI75 significantly higher 5 mg, 10 mg, ETN v PBO (40% 64% 59% v 6%). Non-inferiority for 10 mg dose v ETN.
OPT Pivotal 2 [27,32]	cs-therapy-IR (n = 666) 52-weeks	Continue tofa or withdraw to PBO.	Significantly greater proportion maintained PASI75 v withdrawn to PBO. On retreatment, 37% and 61% who relapsed, achieved PASI 75 by 16 w.

DMARD = Disease modifying antirheumatic therapy; MTX = Methotrexate; ADA = Adalimumab; ETN = Etanercept; PBO = Placebo; IR = Inadequately response. ACR = Improvement of 20%, 505 or 70% in tender/swollen joints, + 3/5 criteria: patient or physician global assessment, functional ability, visual analog pain scale, ESR or CRP; HAQ-DI = Health Assessment Questionnaire Disability index; DAS < 3.2 = Disease activity score less than 3.2 indicating low disease activity DAS < 2.6 = Disease activity score less than 2.6 indicating remission. PASI75 = 75% reduction in the Psoriasis Area and Severity Index score; LEI = Leeds Enthesitis Index score.

ii) Psoriatic arthritis (PsA)

There are no phase II clinical trials specific to PsA. The early-phase studies were mainly conducted in RA, inflammatory bowel disease and psoriasis. Two phase III trials evaluated the efficacy and safety in PsA: OPAL Broaden and OPAL Beyond [26,27] (Table 1). OPAL Balance is a long-term extension (LTE) study of OPAL Broaden and Beyond. Initial results from 24 months demonstrate maintenance of efficacy over time [28]. With the caveat that these trials were not designed for efficacy in skin disease, tofacitinib appears inferior to biologics with lower Psoriasis Area and Severity Index (PASI) 75 responses (40% versus 60%) [29]. While there have been comparatively fewer phase III RCTs for tofacitinib in PsA, the results are consistent to those seen in RA. Long-term extension (LTE) data is reassuring, with no new safety signals.

iii) Psoriasis

Tofacitinib was effective in the treatment of moderate‐to‐severe plaque psoriasis in one phase IIb study [30] and two-phase III studies; Oral-treatment Psoriasis Trial Pivotal 1 and Pivotal 2 [31,32] (Table 1). Although tofacitinib is more effective in psoriasis than traditional medications (e.g. methotrexate), at the 5 mg dose licenced for use in RA it demonstrates inferior efficacy compared to biologics (anti-TNF, ustekinumab and secukinumab). It is currently not licenced for use by the FDA or EMA.

iv) Ankylosing spondylitis (AS)

Tofacitinib has been evaluated in one phase II study in AS. In this 16-week dose-ranging trial in biologic naïve patient with Bath Ankylosing Spondylitis Disease Activity Index (BASDAI) score ≥4 and back pain score ≥4, 5 mg tofacitinib was associated with a significantly higher ASAS20 response rate at week 12 than placebo (81% vs 41%). Secondary outcomes including ASAS40 and BASDAI50 response were met. [33]. Approximately one-third of tofacitinib-treated patients experienced clinically meaningful reductions in spinal MRI inflammation [34]. Tofacitinib is being evaluated in 1 phase III trials in AS (estimated to complete in 2020).

### 3.2 Baricitinib

Baricitinib (Olumiant ®) was developed by Eli Lily and approved by the EMA in 2017. The FDA approved the 2 mg dose in 2018 but declined approval of the 4 mg dose citing safety concerns.

#### 3.2.1 Pharmacokinetics

Baricitinib inhibits JAK1 and JAK2, and to a much lesser extent TYK2. It is considered a JAK3 sparing agent with a 100-fold selectivity for JAK1 and JAK2 [8]. In vitro studies have demonstrated that baricitinib inhibits IFN-γ and IL-6 via JAK1-JAK2, IL-12/23 via JAK2-TYK2 and erythropoietin and granulocyte-macrophage colony-stimulating factor via JAK2-JAK2 [35].

Baricitinib undergoes renal excretion through glomerular filtration and active secretion. Less than 10% is metabolised, mediated by CYP3A4. [11,12] Exposure is generally not affected by co-administration of CYP3A4 inducers or CYP3A4 / CYP2C19 inhibitors. A reduced dose of 2 mg is recommended for patients with a creatinine clearance between 30–60 ml s/minute or those taking OAT3 inhibitors such as probenecid [11].

#### 3.2.2 Trial evidence across diseases

i) Rheumatoid arthritis

Baricitinib has demonstrated significant efficacy in phase II and III RCTs. The development program includes one phase I, three phase II and four phase III trials: RA Begin, Build, Beam, and Beacon [[36], [37], [38], [39]] (Table 2). Patients completing phase III RCTs entered LTEs RA-Beyond and RA-Balance. A sub-study within RA-Beyond examined dose reduction from 4 mg to 2 mg in patients who achieved clinical disease control with 4 mg. Dose tapering resulted in a statistically significant, if modest increase in disease activity, although most patients could retain disease control or regain it by returning to the 4 mg dose [40]. As baricitinib has only recently been approved in the EU and US, less data is available on its use in the ‘real world’. The baricitinib RCTs suggested a potential safety signal, which led to a delay in licensing in North America (only the 2 mg dose is licenced). In the EU both doses are licenced for use in RA. Registry data will be crucial in further characterising safety signals.

**Table 2:** Baricitinib published pivotal phase III RCTs.

**Baricitinib Pivotal Phase III RCTs**			
**Rheumatoid arthritis**			
RA-Begin [36]	DMARD-naive (n = 588) 52-week	Bari 4 mg +/- MTX, or PBO with MTX	At 24 w; ACR20 non-inferior bari monotherapy Vs MTX (77% v 62%). Improved ACR50, ACR70, DAS28‐CRP, HAQ-DI & SDAI in both bari arms. Significantly less radiographic progression on in bari/MTX v MTX.
RA-Build [37]	DMARD-IR (n = 684) 24-week	Bari 2 mg, 4 mg or PBO, with csDMARDs	At 12 w; ACR20 significantly higher 4 mg v PBO (62% v 39%). Improved DAS28, SDAI, HAQ-DI, & radiographic progression at both doses.
RA-Beam [38]	DMARD-IR (n = 1307) 52-week	Bari 4 mg, ADA 40 mg or PBO, with MTX	At 12 w; ACR20 significantly higher 4 mg, ADA v PBO (70%, 61% v 40%). Non-inferior bari V ADA (margin of 12%) -> considered superior (P = 0.01). Improved DAS28, SDAI, HAQ-DI, & radiographic progression.
RA-Beacon [39]	Biologic-IR (n = 527) 24-week	Bari 2 mg, 4 mg or PBO, with csDMARDs	At 12 w; ACR20 significantly higher 4 mg v PBO (55% v 27%). Improved DAS28 & HAQ-DI, but not SDAI remission.

DMARD = Disease modifying antirheumatic therapy; MTX = Methotrexate; ADA = Adalimumab; ETN = Etanercept; PBO = Placebo; IR = Inadequately response. ACR = Improvement of 20%, 505 or 70% in tender/swollen joints, + 3/5 criteria: patient or physician global assessment, functional ability, visual analog pain scale, ESR or CRP; HAQ-DI = Health Assessment Questionnaire Disability index.

ii) Psoriasis

Baricitinib was effective in the treatment of moderate‐to‐severe plaque psoriasis in one phase IIb dosing study. Significantly more patients receiving baricitinib 8 mg and 10 mg attained PASI‐75 compared to placebo [43% and 54% vs. 17% respectively). PASI‐50 response was significantly greater for all baricitinib groups except the 2 mg [41]. As seen with tofacitinib, at the dose licenced for RA baricitinib is inferior to biologics, requiring higher doses to effectively target skin disease.

There are currently no trial data to support the use of baricitinib in PsA or AS.

### 3.3 Use of JAK inhibitors in the management of rheumatoid arthritis

In clinical practice, JAKi are commenced after patients have failed to respond to conventional synthetic disease modifying anti-rheumatic drugs (csDMARDs) such as methotrexate. However, they are often used after biologics have been trialled. This is due to clinical inertia. The JAKi are the latest drugs to come to market, and as such there is less safety data compared to biologics.

The 2017 European guidelines for the management of RA recommend the addition of a biologic or JAKi after failing to respond to the first csDMARD strategy and if poor prognostic factors are present [42]. A small preference is given to biologics due to the availability of long-term safety data. A similar approach was previously used in justifying the use of TNF-inhibitors as the preferred first-line biologic over other biologics due to a long-term efficacy and safety data from registries. The 2015 American guidelines include tofacitinib alone as the only FDA-approved JAKi at the time of publication [43]. If disease activity persists despite first csDMARD strategy, the guidelines recommend without preference, either the use of combination csDMARD, the addition of a biologic or the addition of tofacitinib. Hierarchy of choice is not ranked as there is no evidence of superiority from direct comparison trials. If disease activity remains high despite a biologic, a second-line biologic is recommended over tofacitinib. This is justified by longer‐term safety data and clinical experience, without differences in clinical efficacy.

JAKi are efficacious in both patients who are biologic-naive and those who had failed previous biologics, although response rates are numerically greater in the biologic-naïve group [23,44]. This contrasts with earlier biologic trial data, in which the clinical response was significantly lower when a second biologic was used after a prior failure. The improved efficacy of JAKi in patients who have failed a biologic may relate to changes in the definition of biologic failure and a lower threshold for switching therapies. This has resulted in a population who may align more with a biologic naive cohort.

### 3.4 Biomarkers for JAK inhibitors response in rheumatoid arthritis

Identifying factors that predict the likelihood of clinical response may be of benefit in treatment decision making. Trial data has demonstrated that CRP predicts response to JAKi therapy, with a high baseline CRP associated with greater treatment efficacy [45], The inherent problem is that secretion of CRP is largely IL-6 dependant. JAKi interrupt this pathway, leading to a fall in CRP which does not necessarily reflect a decrease in disease activity. RCTs have actively recruited patients with elevated CRP levels and the predictive value of CRP has not yet been demonstrated in the real world. Anticyclic citrullinated peptide (ACPA) is also associated with treatment response to JAKi [46]. Although serostatus does not markedly influence treatment outcomes, a negative ACPA is associated with an increased risk of treatment discontinuation [47].

## 4 JAK inhibitors in development

Next-generation JAK inhibitors have been designed with a view to improve selective affinity for one or more of the four JAK enzymes. The principle aim of these agents is to reduce non-selective pan JAK inhibition in the hope that this will lessen unwanted adverse effects without a decline in clinical efficacy. These agents are yet to be licensed in Europe or North America.

### 4.1 Upadacitinib

Upadacitinib (ABT-494) was developed by AbbVie [48].

#### 4.1.1 Pharmacokinetics

Upadacitinib is a selective JAK1 inhibitor, with 74 and 58 -fold selectivity for JAK1 over JAK2 and JAK3 respectively [49]. This is due to its ability to bind JAK1 at two separate sites. In vitro research suggests that JAK1 inhibition might be largely responsible for the in vivo efficacy of JAK inhibitors in immune-inflammatory diseases [50]. Upadacitinib exposure is weakly decreased with strong inhibition of CYP3A4 and moderately increased with broad CYP induction [51]. Approximately 20% is eliminated by the kidneys. [52].

#### 4.1.2 Trial evidence across diseases

i) Rheumatoid arthritis

In RA, upadacitinib has been evaluated in two phase II dose ranging studies: BALANCE I (in TNF inadequate responders) and BALANCE II (in methotrexate inadequate responders), in which a significant and rapid dose-response were seen for ACR20, 50 and 70 responses [49,53]. Upadacitinib is currently being evaluated in six phase III RCTs, four of these are complete with published data: SELECT-Next, Beyond, Early and Monotherapy [[54], [55], [56], [57]] (Table 3). Two phase III RCTs are ongoing, SELECT-Compare examines upadacitinib versus adalimumab versus placebo in methotrexate inadequate responders (projected to complete in 2020) and SELECT-Choice is a non-inferiority study examining upadacitinib versus abatacept in bDMARD inadequate responders (estimated to complete in 2021).

**Table 3:** Upadacitinib published pivotal phase III RCTs.

**Upadacitinib Pivotal Phase III RCTs**			
**Rheumatoid arthritis**			
SELECT-Next [54]	DMARD-IR (n = 661) 12-week	UPA 15 mg, 30 mg or PBO, with csDMARDs	At 12 w; significantly higher 15 mg, 30 mg v PBO ACR20 (64%, 66% v 39%) & DAS28-CRP≤3.2 (48%, 48% v 17%). Improved HAQ-DI, FACIT & stiffness.
SELECT-Beyond [55]	bDMARD-IR (n = 498) 12-week	UPA 15 mg, 30 mg or PBO, with csDMARDs	At 12 w; significantly higher 15 mg, 30 mg v PBO ACR20 (65%, 56% v 48%) & DAS28-CRP≤3.2 (43%, 42% v 14%). Improved ACR50 (& ACR70 t 30 mg only). At 24 w, responses were similar PBO->UPA at 12 w vs assigned UPA at B/L.
SELECT-Early* [56]	MTX naive (n = 945) 24-week	UPA 15 mg, 30 mg or MTX	At 12 w; significantly higher 15 mg, 30 mg v PBO ACR50 (52%, 56% v 28%) & DAS28-CRP≤2.6 (48%,50% v 19%). Less radiographic progression both doses.
SELECT-Monotherapy* [57]	DMARD/biologic-IR (n = 648)	UPA 15 mg, 30 mg or PBO	At 14 w; significantly higher 15 mg, 30 mg v PBO ACR20 (68%, 71% v 41%) & DAS28-CRP≤3.2 (45%,53% v 19%). Improved ACR50, ACR70 & DAS28 < 2.6.

DMARD = Disease modifying antirheumatic therapy; MTX = Methotrexate; ADA = Adalimumab; ETN = Etanercept; PBO = Placebo; IR = Inadequately response. ACR = Improvement of 20%, 505 or 70% in tender/swollen joints, + 3/5 criteria: patient or physician global assessment, functional ability, visual analog pain scale, ESR or CRP; HAQ-DI = Health Assessment Questionnaire Disability index.

* Abstract only.

ii) Psoriatic arthritis

Upadacitinib is being evaluated in two phase III trials in PsA. SELECT-PsA1 in DMARDs inadequate responders, comparing upadacitinib to placebo and to adalimumab and SELECT-PsA2 in bDMARDs inadequate responders comparing upadacitinib to placebo (both estimated to complete in 2023).

iii) Ankylosing spondylitis

Upadacitinib is being evaluated in one phase II/III trial in AS. SELECT-Axis 1 is comparing upadacitinib to placebo in biologic naïve patients (estimated to complete in 2020).

### 4.2 Filgotinib

Filgotinib (GLPG0634) has been co-developed by Galapagos and Gilead Sciences [48].

#### 4.2.1 Pharmacokinetics

Filgotinib is also a selective JAK1 inhibitor. In whole blood assays it shows a 30-fold selectivity for JAK1 over JAK2-dependent signalling [58]. Preclinical studies demonstrated that filgotinib forms an active metabolite that exhibits a similar JAK1 selectivity profile as filgotinib, albeit less potent. This metabolite contributes to the relatively long duration of JAK1 inhibition following filgotinib dosing [59]. Neither filgotinib nor its active metabolite inhibit or induce CYP activity at clinically relevant concentrations, a potentially attractive feature for patients on multiple medications.

#### 4.2.2 Trial evidence across diseases

i) Rheumatoid Arthritis

In RA, Filgotinib has been investigated in two phase IIb studies. DARWIN-1 was a 24-week trial in MTX inadequate responders with background MTX. At week 12, ACR 20 responses were significantly higher with filgotinib 100 and 200 mg, with no difference between once-daily and twice-daily regimens. Onset of action was rapid and dose-dependent responses were observed for most efficacy endpoints [60]. DARWIN-2 was a 24-week trial of filgotinib monotherapy in MTX inadequate responders. At week 12, significantly more patients receiving filgotinib at any dose achieved ACR20 responses versus placebo (≥65% vs 29%). In both studies there were statistically significant improvements in ACR50, ACR70, DAS28-CRP, CDAI and HAQ-DI [61].

Filgotinib is currently being evaluated in three phase III RCTs in RA and one LTE. FINCH 1 is a 52-week RCT in MTX inadequate responders, comparing filgotinib plus MTX versus placebo and adalimumab. FINCH 2 is a 24-week RCT in DMARD inadequate responders taking csDMARDs. It completed in June 2018, although no results have been published at time of writing. FINCH 3 is a 52-week RCT in MTX-naïve patients examining filgotinib in combination with MTX, as well as monotherapy.

ii) Psoriatic arthritis

Filgotinib is being evaluated in one phase II trial in PsA. The EQUATOR trial in DMARD inadequate responders reported a statistically significant improvement in ACR20 at week 16 with filgotinib and in combination with csDMARD compared to placebo (80% v 33%). Similar findings were seen for ACR50 and ACR70 responses (treatment difference 33% and 17% respectively) [62].

iii) Ankylosing spondylitis

Filgotinib has been evaluated in one phase II trials for AS. The TORTUGA trial reported a statistically significant change from baseline in ankylosing spondylitis disease activity score (ASDAS) at week 12 compared to placebo. Significant differences were reported in ASAS20 (76% v 40%) and ASAS40 (38% v 19%) and in a change in BASDAI from baseline [63].

## 5 JAK inhibitors in early development and those that have been discontinued

Table 4 summaries the JAK inhibitors that are early in trial development including possible breakthrough agents and those in whom development has been discontinued.

**Table 4:** JAK inhibitors in early development and those that have been discontinued.

**Compound**	**Company**	**Target**	**Phase**	**Indication**	**Trial number**	**Status**
SHR0302	HengRui	JAK1	I	RA	NCT03254966	
AC430	Ambit	JAK2	I	RA	NCT01287858	
PF-06263276	Pfizer	Pan-JAK	I	Psoriasis	NCT02193815	
Solcitinib (GSK2586184)	GSK	JAK1	II	Psoriasis	NCT01782664	Discontinued
BMS-986165	BMS	TYK2	II	Psoriasis	NCT02931838	
Lestaurtinib (CEP-701)	Cephalon	JAK2 (FLT3/TrkA)	II	Psoriasis	NCT00236119	
PF-06651600	Pfizer	JAK3	II	RA	NCT02969044	
Ruxolitinib	Incyte	JAK1 /JAK2	II	RA	NCT00550043	
				Psoriasis	NCT00820950 NCT00778700 NCT00617994	
Itacitinib (INCB039110)	Incyte	JAK1/JAK2	II	RA	NCT01626573	Discontinued
				Psoriasis	NCT01634087	
PF-04965841	Pfizer	JAK1/TYK2	II	Psoriasis	NCT02969018	
Peficitinib (ASP015 K)	Astellas	Pan JAK	II	Psoriasis	NCT01096862	
			III	RA	NCT01638013 NCT02305849 NCT02308163	
Decernotinib (VX509)	Vertex	JAK3	II & III	RA	NCT01830985 NCT01590459 NCT01052194	Discontinued
Abrocitinib (PF-04965842)	Pfizer	JAK1	III	Psoriasis	NCT02201524	Discontinued

GSK: GlaxoSmithKline; HengRui: Jiangsu Hengrui Medicine; BMS: Bristol-Myers Squibb.

*i) Discontinued agents:* The efficacy, safety and tolerability of solcitinib was evaluated in patients with moderate-to-severe plaque psoriasis [64]. However, during a phase II trial evaluating solcitinib in SLE, severe adverse events (elevated liver enzymes, leading to DRESS syndrome) and the discovery of a drug interaction with statins necessitated early termination of the trial [65], and led to the discontinuation of further drug development. Decernotinib is a selective JAK3 inhibitor that demonstrated comparable efficacy to tofacitinib in RA [66]. However, it was reported to cause neutropenia and, as a potent inhibitor of CYP3A4, was likely to contribute to multiple drug interactions. As such the manufacturer decided not to proceed with development of this drug for RA [58].

*ii) Breakthrough agents:* Peficitinib inhibits JAK1, JAK2, JAK3, and TYK2 activities with moderate selectivity for JAK3 inhibition. The efficacy of peficitinib for the treatment of RA has been investigated in phase II trials, with similar efficacy as seen with other JAKi [[67], [68], [69]]. PF-06651600 targets JAK3 and was developed by modifying the structure of tofacitinib to allow irreversible covalent binding and optimize selectivity. It is being evaluated in the treatment of RA [70]. Topical ruxolitinib has demonstrated efficacy in psoriasis, but as with tofacitinib the improvement was not sustained after discontinuation [71]

## 6 Safety

Returning to small molecules use after years of biological therapy requires careful consideration of their safety. Unlike biologics, JAKi’s demonstrate a dose-proportional pharmacokinetic profile. At higher doses they exhibit ‘pan-JAK’ inhibition with resultant off target effects [7]. The therapeutic window is controlled by hepatic metabolism facilitated by cytochrome P450 and renal clearance through glomerular filtration and active secretion. This introduces the risk of toxicity when co-administered with potent CYP3A4 (e.g., ketoconazole) or CYP2C19 inhibitors (e.g. fluconazole) or in patients with severe renal impairment. Current recommendations advise dose reduction in such circumstances. [11,12]. It is clear that further work is required to fully characterize the safety profile of JAKi. Registry data will play a prominent role, assessing safety and efficacy in a more heterogenous population. Prescribing clinicians should be vigilant and keep an open mind regarding novel adverse events.

### 6.1 Infection

As JAK inhibition results in the suppression of multiple integral elements of the immune response, infection represents a major concern [6]. The introduction of JAKi was initially overshadowed by concerns of opportunistic infection observed at higher doses. As the phase III trials have emerged and LTE data has been evaluated, the absolute risk of serious adverse events appears comparable to biologics.

Pooled data from tofacitinib studies with 19,406 patient years demonstrated a serious infection incidence rate of 2.7 per 100 patient years [72]. Glucocorticoids, baseline lymphopenia, line of therapy (3^rd^ line vs 2^nd^) and geographical region (Asia, Europe, and Latin America versus USA and Canada) are associated with greater risk [72]. Similar rates of serious infection were reported with baricitinib. A pooled analysis including 6637 patient years reported an incidence rate of 2.9 per 100 patient years [73]. The risk of serious infections is comparable to published rates for biologics. A meta-analysis reported a rate of 3.02 and 2.50 in tofacitinib RCTs and LTEs, which was similar to rates seen with biologics (range 3.04 to 5.45) [74].

The most recognized infectious complication with JAKi has been the reactivation of varicella zoster virus, with incidence rates of 4.4 per 100 patient-years with tofacitinib [75] and 3.2 with baricitinib [73]. These rates are substantially higher in Asia (7.7 with tofacitinib) [75]. An observational analysis using US health plan data reported an approximate doubling in the rate of herpes zoster with tofacitinib compared to biologics (adjusted hazard rate 2.01 compared to abatacept) [76]. The highest rates are seen in older patients with co-prescription of glucocorticoids or MTX, and in those from Japan or Korea [75]. There are very few cases of multi-dermatomal or disseminated herpes, and no cases of visceral disease or death [75].

Tuberculosis was reported with both tofacitinib and baricitinib. With tofacitinib there were 26 cases identified from RCTs and LTEs, of which 20 occurred with the 10 mg dose, and all but two cases had negative screening at trial entry [77]. With baricitinib there were 10 cases, all of which occurred in endemic areas [73]. Screening for tuberculosis (i.e. with Interferon Gamma Release Assay, IGRA) is recommended across the JAK class.

### 6.2 Malignancy

A theoretical concern exists regarding the risk of malignancy. JAKi’s block interferon signaling, a central coordinator in tumour surveillance, and NK cells known for their ability to kill tumour cells [78]. This cancer signal may have a longer latency than observed with other safety outcomes. Pooled data from tofacitinib studies with 19,406 patient years recorded 173 malignancies and 118 non-melanoma skin cancers (NMSC) in 6194 patients [72]. The most common cancers were lung (n = 32) followed by breast (n = 25) and lymphoproliferative (n = 19) [72]. The standardized incidence ratios for all malignancies and for NMSC were within the expected range seen in patients with moderate-to-severe RA, and the rate remained stable over time [79]. Fewer data are available for baricitinib. A safety analysis of eight RCTs and one ongoing LTE study with 6637 patient years recorded 52 malignancies and 24 NMSC in 3492 patients (46% had exposure data for less than two years). Although reassuring, long term experience with these agents are limited in comparison to biologics such as TNF inhibitors. Post-marketing surveillance (e.g. drug registries) is essential in evaluating the risk of malignancy with JAKi [6].

### 6.3 Venous thromboembolism (VTE)

Analysis of VTE across tofacitinib studies in RA, psoriasis, PsA and ulcerative colitis showed no evidence of an increased risk [80]. At three months follow up there were two VTEs in the placebo arm and none in the treatment arms. In total there were three deep vein thromboses (two in RA, and one in PsA) and five pulmonary emboli (PE) (all in RA). In general, the numbers of VTEs were small, with a surprising higher rate of PE than DVT, which may suggest underreporting of DVTs. The incidence rates for PE in the RA RCTs were similar to those reported with biologics [80].

The baricitinib studies identified an imbalance in the number of VTEs. An analysis of pooled data in 3492 baricitinib-RA treated patients recorded 31 VTEs. At six months follow up, six patients taking with baricitinib 4 mg had a VTE. There were no VTEs in the placebo arm. All patients with VTE had multiple risk factors and the rates remained stable over time. At longer exposure, the overall incidence rate for DVT/PE was 0.5 per 100 patient years. The rate was comparable between doses (0.5 vs 0.6 in 2 mg and 4 mg respectively) [73,81]. The published incidence rate for DVT/PE in the RA population is 0.3 to 0.8 per 100 patient years [82]. The potential signal around VTE that have been observed in the data is unconvincing at present. Clinically, it would be wise to be cautious in patients with risks factors for VTE, and to consider other therapies first. However, it would be wrong to suggest that there is conclusive evidence of a VTE risk with baricitinib therapy.

### 6.4 Lipids

Hypercholesterolemia and changes in lipoprotein composition have been observed with JAK inhibitors.

It remains unclear if or how inhibition of the JAK pathway influences lipid structure and function. There are similarities to the lipid raising effects seen with the IL-6 inhibitor tocilizumab, suggesting the mechanism may lie in blockade of the IL-6 pathway. Invitro studies have demonstrated that tofacitinib reduces cholesterol ester catabolism [83] and increases lipid release from macrophages through its actions on reverse cholesterol transport [84].

In tofacitinib RA studies a 16–30% dose-dependent increase in HDL and LDL has been reported [85]. Levels increased within one month and then plateaued. A dose-dependent increase in HDL, LDL and triglycerides was also observed with baricitinib. Despite these changes the LDL:HDL ratio remained stable. With both agents, alterations in lipids correlated with improvements in RA disease activity [86]. Patients with RA demonstrate abnormal lipids profiles as a direct consequence of their disease. It is postulated that JAKi restore lipid balance. However, this seem unlikely as data from ulcerative colitis trials, where there is no established link with lipid metabolism, report unfavourable changes in lipid profiles with JAKi. Although elevations in LDL levels is concerning, this did not translate into increased cardiovascular events during the RCTs or LTEs. It is important that we don’t assume that the elevation in lipids can be ignored. Patients who demonstrated changes were treated with statins, with LDL levels reversing in response to therapy [87]. It may be more appropriate to conclude that in patients receiving JAKi, who are treated for hypercholesteremia, there is no added increase in cardiovascular risk. Longer-term data are available with tocilizumab, which reassuringly has not demonstrated an associated risk of cardiovascular disease [88].

### 6.5 Gastrointestinal perforation

An elevated incidence of lower intestinal perforations has been reported with JAKi, similar to that seen with tocilizumab [89]. There were 22 GI perforations from the tofacitinib pooled data with 19 406-person years of drug exposure, with an incidence rate of 0.11 per 100 patient years [72]. A study using health plan data reported a two-fold risk of GI perforation among tofacitinib users compared to those receiving TNFi, although this was not statistically significant [90]. A baricitinib safety analysis with 6637-patient years reported three GI perforations, with an incidence rate of 0.05 per 100 patient years [91]. As seen with tocilizumab the risk was greatest in patients with known diverticular disease. Data from tocilizumab studies has highlighted that patients present atypically with subtle clinical signs and a blunted inflammatory response [92]. It would be prudent to follow similar caution with patients receiving JAKi.

### 6.6 Pregnancy

Small molecule JAKi potentially cross the placenta. Due to the unknown risks to mother and child, RCTs excluded pregnant patients and required the use of effective contraception by all women of child-bearing potential. Nonetheless pregnancies did occur and outcomes recorded where possible. Of the 9815 patients enrolled in the tofacitinib RCTs, 47 pregnancies (31 with RA and 16 with psoriasis) were reported. There were 25 healthy new-borns, no foetal deaths, seven spontaneous abortions, eight medical terminations and one congenital malformation. The frequency of abortions and congenital malformation were consistent with the background population risk [93]. Despite these reassuring data, JAKi are not licensed for use in pregnancy.

## 7 Conclusion

The past decade has witnessed an explosion in trial data on JAKi. These drugs have the potential to be a game changer in the management of rheumatic diseases. They are advantageous in their oral availability and rapid onset of action. The efficacy demonstrated by first generation agents is on par with existing biologics, whilst emerging data with next-generation JAKi may suggest even greater success. These drugs have important dose-proportional pharmacokinetic profiles and key safety signals e.g. viral infection (shingles) which will need careful management in clinical practice. As our understanding of the implications of JAK selectivity grows, we move one step closer towards the world of personalised medicine. It is evident that JAKi are revolutionising the therapeutic armamentarium in inflammatory driven pathologies. Clinicians must now consider the place of these drugs in the management of rheumatic disease as they appear destined to take centre stage.

## Disclosure statement

J.B.G. has received honoraria for speaking or attending conferences from Pfizer, Bristol-Myers Squibb, UCB and Celgene.

## Funding statement

This work was supported by the Medical Research Council in the form of a Clinical Training Research Fellowship to KB (<GN1>CTRF- MR/R001332/1)</GN1>. No specific funding was received from any bodies in the public, commercial or not-for-profit sectors to carry out the work described in this manuscript.

## Declaration of Competing Interest

None declared by authors.
