# Corrigendum to “The new entries in the therapeutic armamentarium: The small molecule JAK inhibitors” [Pharmacol. Res. 147 (2019) 104392]

**DOI:** 10.1016/j.phrs.2020.104634

**Published:** 2020-03

**Authors:** Katie Bechman, Mark Yates, James B. Galloway

**Affiliations:** Centre for Rheumatic Diseases, Kings College London, United Kingdom

In this article two typographical errors have been identified.

The sentence "At 12 w; significantly higher 15 mg, 30 mg v PBO ACR20 (65 %, 56 % v 48 %) in Table 3 is now corrected into "At 12 w; significantly higher 15 mg, 30 mg v PBO ACR20 (65 %, 56 % v 28**%**)”.

The study with Abrocitinib (PF-04965842) mentioned in Table 4 was a phase II not a phase III study as erroneously indicated.

The authors would like to apologise for any inconvenience caused.

